# Army Medical Appointments

**Published:** 1859-07

**Authors:** 


ARMY MEDICAL APPOINTMENTS.—The Army Medical Board recently held its meeting in this city, and made the following appointments to the Army Medical Staff:—1. George Sackley, M.D., of New York; 2. D. C. Peters, M.D., of New York; 3. C. H. Alden, M.D., of Pennsylvania.

Assistant Surgeons A. B. Hasson and T. Letherman, having been examined, were found qualified for promotion.

